# ACC2 Is Expressed at High Levels Human White Adipose and Has an Isoform with a Novel N-Terminus

**DOI:** 10.1371/journal.pone.0004369

**Published:** 2009-02-03

**Authors:** John C. Castle, Yoshikazu Hara, Christopher K. Raymond, Philip Garrett-Engele, Kenji Ohwaki, Zhengyan Kan, Jun Kusunoki, Jason M. Johnson

**Affiliations:** 1 Rosetta Inpharmatics LLC, Seattle, Washington, United States of America; 2 Metabolic Disorder Research, Tsukuba Research Institute, Banyu Pharmaceutical Co., Ltd., Tsukuba, Ibaraki, Japan; Dr. Margarete Fischer-Bosch Institute of Clinical Pharmacology, Germany

## Abstract

Acetyl-CoA carboxylases ACC1 and ACC2 catalyze the carboxylation of acetyl-CoA to malonyl-CoA, regulating fatty-acid synthesis and oxidation, and are potential targets for treatment of metabolic syndrome. Expression of ACC1 in rodent lipogenic tissues and ACC2 in rodent oxidative tissues, coupled with the predicted localization of ACC2 to the mitochondrial membrane, have suggested separate functional roles for ACC1 in lipogenesis and ACC2 in fatty acid oxidation. We find, however, that human adipose tissue, unlike rodent adipose, expresses more ACC2 mRNA relative to the oxidative tissues muscle and heart. Human adipose, along with human liver, expresses more ACC2 than ACC1. Using RT-PCR, real-time PCR, and immunoprecipitation we report a novel isoform of ACC2 (ACC2.v2) that is expressed at significant levels in human adipose. The protein generated by this isoform has enzymatic activity, is endogenously expressed in adipose, and lacks the N-terminal sequence. Both ACC2 isoforms are capable of *de novo* lipogenesis, suggesting that ACC2, in addition to ACC1, may play a role in lipogenesis. The results demonstrate a significant difference in ACC expression between human and rodents, which may introduce difficulties for the use of rodent models for development of ACC inhibitors.

## Introduction

Acetyl-CoA carboxylase alpha (ACC1) and beta (ACC2) catalyze the carboxylation of acetyl-CoA to malonyl-CoA. Malonyl-CoA is a substrate for fatty acid synthase and also inhibits carnitine palmitoyltransferase-1, such that malonyl-CoA is a key molecule regulating both the biosynthesis and oxidation of fatty acids. Thus ACC1 and ACC2 are key regulators of the fatty-acid synthesis and oxidation pathways [1]–[5].

In mammals, both of ACC1 and ACC2 are multifunctional enzymes, containing biotin carboxyl carrier, biotin carboxylase, and carboxyl transferase domains all within a single polypeptide chain (Supplemental Figure S1). Although ACC1 and ACC2 share over 70% protein sequence identity and have the same enzymatic activity, they are believed to have distinct cellular roles. Indeed, while mouse ACC1^−/−^ mutants are embryonic lethal [6], [7], mutant ACC2^−/−^ mice have a normal lifespan but higher fat oxidation rate in muscle and adipose tissues and are resistant to diet-induced obesity [8]–[10], observations which have made ACC2 an attractive target for treatment of obesity and type 2 diabetes [7], [11]–[13]. ACC1 is cytosolic and believed to be the primary acetyl-CoA carboxylase (ACC) involved in *de novo* fatty-acid synthesis. This is supported by observations of high ACC1 expression in rat and mouse lipogenic tissues [14], [15]. In contrast, ACC2 has been reported to regulate fatty-acid oxidation through malonyl-CoA mediated inhibition of carnitine palmitoyltransferase-1 [14]–[19]. Physiological studies have also linked ACC2 expression in muscle to physical exercise, corroborating a link between ACC2 and fatty-acid oxidation [20]–[22]. Immunofluorescence microscopy studies suggest that ACC2 is preferentially localized to the mitochondria, possibly due to the unique ∼220 amino acid N-terminus of ACC2 [18]. This N-terminus includes a leader sequence of ∼20 hydrophobic residues that has been hypothesized to be responsible for mitochondrial localization and thus the functional difference between two genes [15].

Arguments against a lipogenic role for ACC2 are based primarily on preferential expression of ACC2 in rodents within non-lipogenic tissues. In rat, for example, ACC2 is expressed in muscle, heart, liver, mammary gland, and brown adipose but was not observed in white adipose [14], [23]. In prior human expression studies, ACC2 transcript levels were measured in eight tissues [15], and the highest expression of ACC2 was observed in skeletal muscle. However, ACC2 expression in human adipose tissue was not assayed. In addition, EST sequence databases suggest the existence of novel alternative splice forms [5].

Since ACC inhibition is a candidate treatment for human metabolic syndrome, there is a clear need to characterize ACC expression in human tissues. Here we present RNA and protein evidence that ACC2 is expressed at higher levels in human white adipose than in human skeletal muscle, and that the expression of ACC2 in human white adipose is higher than that of ACC1. Further, we show RNA and protein evidence for a second ACC2 isoform that is expressed in human adipose, has enzymatic and *de novo* lipogenic activity, is endogenously expressed, and has a distinct N-terminus lacking the proposed N-terminal mitochondrial localization sequence. These observations suggest ACC2 may play a role in fatty-acid synthesis, and that there may be molecular differences in fat metabolism between rodents and humans.

## Results

### ACC2 mRNA is present at higher levels in human adipose tissue than ACC1

**Figure 1:**
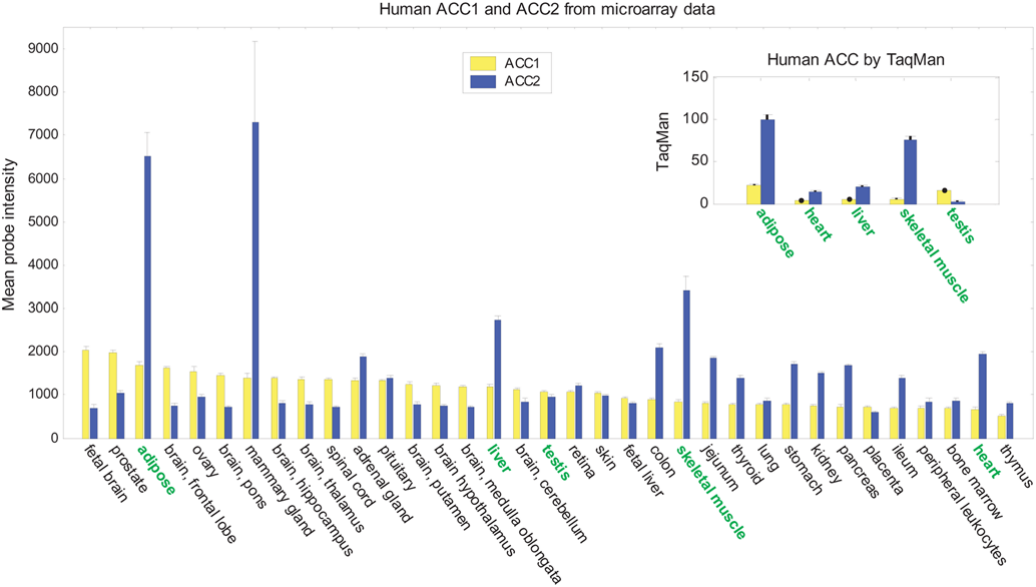
Human ACC1 and ACC2 mRNA expression from microarray and TaqMan (inset) measurements. Microarray data show the average probe intensity from 113 (ACC1) and 120 (ACC2) probes and are ordered based on ACC1 expression. Calibrated TaqMan data were scaled to a maximum of 100.

Expression levels of ACC1 and ACC2 in human tissues, each pooled from multiple donors, were examined using oligonucleotide microarrays [24] and calibrated quantitative real-time RT-PCR (Figure 1). Microarray data were derived from custom microarray patterns, with probes placed in every exon and exon-exon junction, and a random-priming amplification protocol (Materials and Methods). Intensities for ACC1 and ACC2 are the average of over 100 probes per gene. In the tissues examined, ACC1 is expressed at higher levels than ACC2 in brain, prostate, and ovary, whereas ACC2 is expressed at higher levels in adipose, mammary gland, muscle, heart, gastrointestinal tissues, liver, kidney, thyroid, pituitary, and adrenal gland.

We validated these results using calibrated real-time RT-PCR (Taqman). Calibrated Taqman involves construction of a standard curve derived from dilution series measurements, resulting in highly quantitative measurements permitting cross-gene and cross-tissue comparisons. We find the relative expression of ACC1 and ACC2 transcripts measured by microarray and quantitative PCR (Figure 1, inset) are in good agreement. Both datasets clearly show that ACC2 is strongly expressed in adipose tissue, at levels greater than in muscle.

### A transcript variant of ACC2 with a novel N-terminus

**Figure 2:**
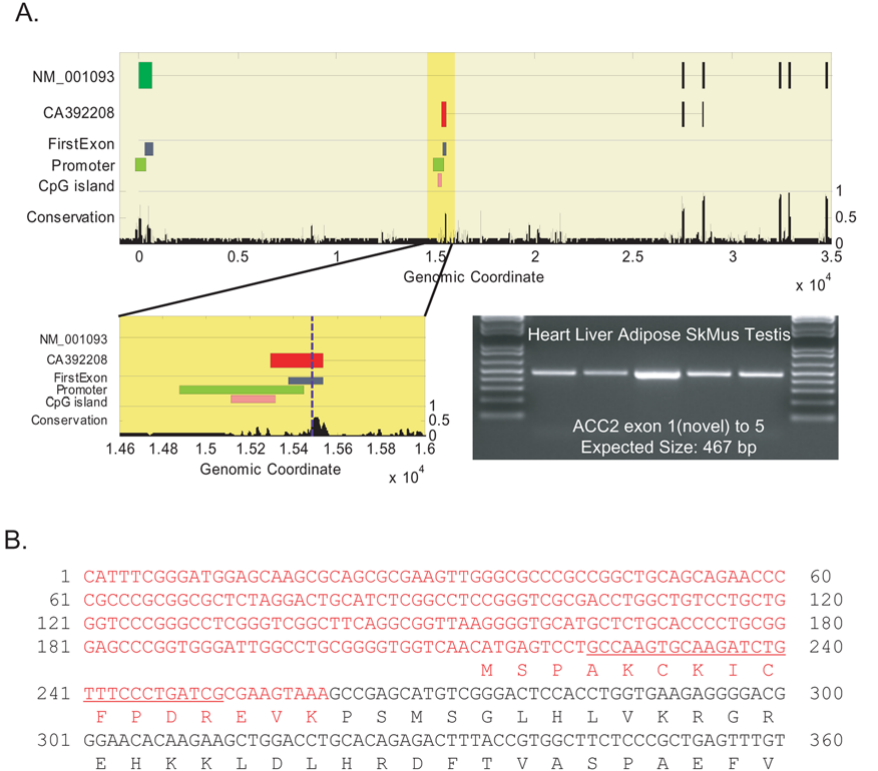
Novel ACC2 isoform. (A) Genomic alignment of the 5′ end of RefSeq transcript NM_001093, human EST CA392208 (exons are shown as boxes), ‘FirstExon’ predictions [33], including the CpG island, promoter region, and first exon location, and conservation score for human, mouse, rat, and chicken [34]. The green and red boxes in the top two rows are the alternative first exons. The vertical, dashed blue line in the zoomed image (lower left panel) shows the predicted start ATG codon. The gel image using RT-PCR primers in novel exon 1 and known exon 5 shows intensity in all tissues tested at the predicted size. (B) The RNA sequence of the novel exon 1 (red), the start of exon 2 (black), and the predicted protein. The first base is the beginning of the aligned mouse EST BB866065. The RT-PCR primer target sequence is underlined.

When mapped to the human genome, three human and mouse ESTs, human CA392208 and mouse BB854145 and BB866065, suggest the existence of an additional transcribed region between the first and second exons of the RefSeq ACC2 isoform (ACC2.v1) NM_001093 (Figure 2A). Other genomic data support this interpretation, including a candidate ATG start codon, high human, rodent and chicken sequence conservation, and the absence of termination codons (Figure 2A, Figure S2). Usage of this ATG codon as start site could generate an in-frame ACC2 protein in which the first 218 amino acids of the known RefSeq protein NP_001084, including the hydrophobic N-terminus, were replaced with a novel 16 amino-acid N-terminus (Figure 2B). This variant, referred to in what follows as ACC2.v2, is less likely to encode a membrane-associated protein because it lacks the putative mitochondrial localization sequence.

### The novel ACC2.v2 transcript is expressed in white adipose

To validate transcription of this computational prediction, we designed RT-PCR primers from the novel exon 1 to the known exon 5. The result shows clear expression of the novel band in several tissues (Figure 2A). Cloning followed by sequencing of this band confirmed the sequence identity, which has been deposited in dbEST as EH093926. This sequence cannot be mapped to any other region of the human genome, including the ACC1 locus, and thus experimentally confirms ACC2.v2 transcription.

### Expression of ACC1 and ACC2 isoforms in human, rat, and mouse tissues

**Figure 3:**
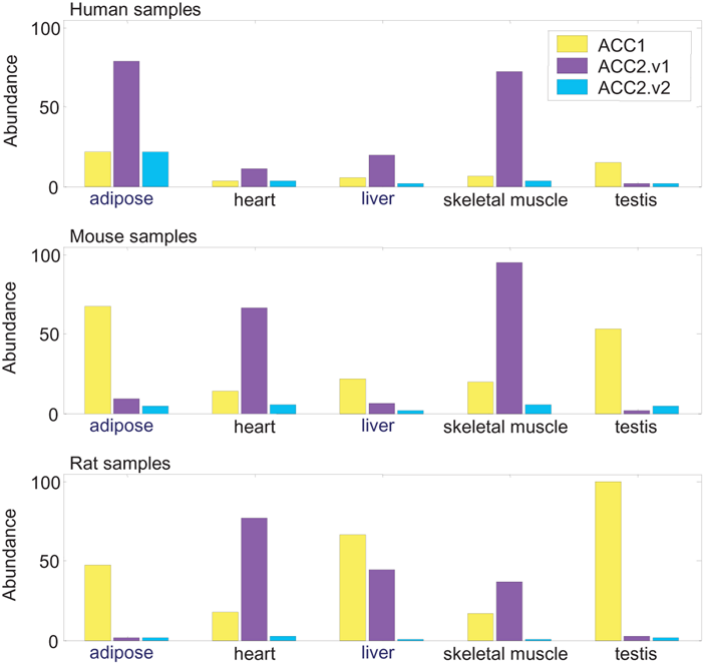
Expression of ACC1 and ACC2 mRNA isoforms in human, mouse, and rat tissues. TaqMan probes were designed to specifically monitor each transcript and were calibrated using standard curves (see Methods). Expression levels were scaled for each species such that the maximum ACC1 or total ACC2 expression (ACC2.v1 plus ACC2.v2) is 100.

The relative abundances of ACC1, ACC2.v1 and ACC2.v2 transcripts were measured in five human tissues using calibrated real-time RT-PCR. As shown in Figure 3 (top), the human ACC2.v2 transcript is less abundant than the known isoform but is significantly expressed in all five tissues tested. The highest absolute expression level of ACC2.v2 is observed in adipose tissue, where the splice variant accounts for over 20% of ACC2 expression, at levels comparable to ACC1 expression. We also measured the levels of ACC1, ACC2.v1, and ACC2.v2 in corresponding mouse and rat tissues (Figures 3, middle and lower). The ratio of ACC1 and ACC2 expression is dramatically different between rodent tissues and human tissues, particularly in the lipogenic tissues adipose and liver. Unlike human tissues, both rat and mouse adipose express ACC2 at low levels, significantly lower than ACC1, and rodent liver contains more ACC1 than ACC2. Furthermore, rodent muscle expresses ACC2 at levels much higher than seen in rodent adipose, different from the pattern in humans. Finally, the ratio of ACC2.v1 to ACC2.v2 transcript expression is similar in rodent and human tissues, further suggesting that the novel ACC2.v2 product is regulated and may be functional.

### ACC1 and ACC2 protein expression in human, rat, and mouse tissue

**Figure 4:**
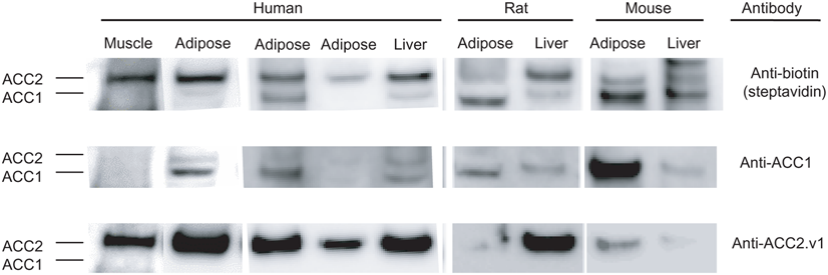
ACC protein expression, measured with three different antibodies: ACC1+ACC2 (top row), ACC1-specific (middle row), and ACC2.v1-specific (bottom row). Each human adipose and liver sample is from a different individual. Streptavidin targets an endogenous biotin molecule within each ACC isoforms, so its intensity reflects molar ratio of each ACC isoform. The ACC2.v1 antibody specifically targets the ACC2.v1 N-terminus and does not detect ACC2.v2 protein.

To determine if these transcript expression patterns reflected enzyme levels, we assayed ACC1 and ACC2 protein levels by Western blot using streptavidin and antibodies specific to the middle of ACC1 and to the N terminus of ACC2.v1 (Figure 4). A single biotin molecule covalently couples to a single ACC molecule, permitting qualitative determination of ACC1 and ACC2 protein expression with streptavidin-HRP (anti-biotin). A bright ACC2 band was observed in the human muscle sample (first column). The three human adipose samples probed all show ACC2 bands and less intense ACC1 bands. Human, rat, and mouse liver showed a mix of ACC1 and ACC2. Rat and mouse white adipose show intensities at the ACC1 band and lower intensities at the ACC2 band. These results, while highly qualitative, suggest that the ACC2 protein is endogenously expressed in human adipose. We also find evidence suggesting the ACC2.v2 protein is endogenously expressed (Supplemental Figure S3).

### ACC2.v2, ACC2.v1, and ACC1 protein activity

**Figure 5:**
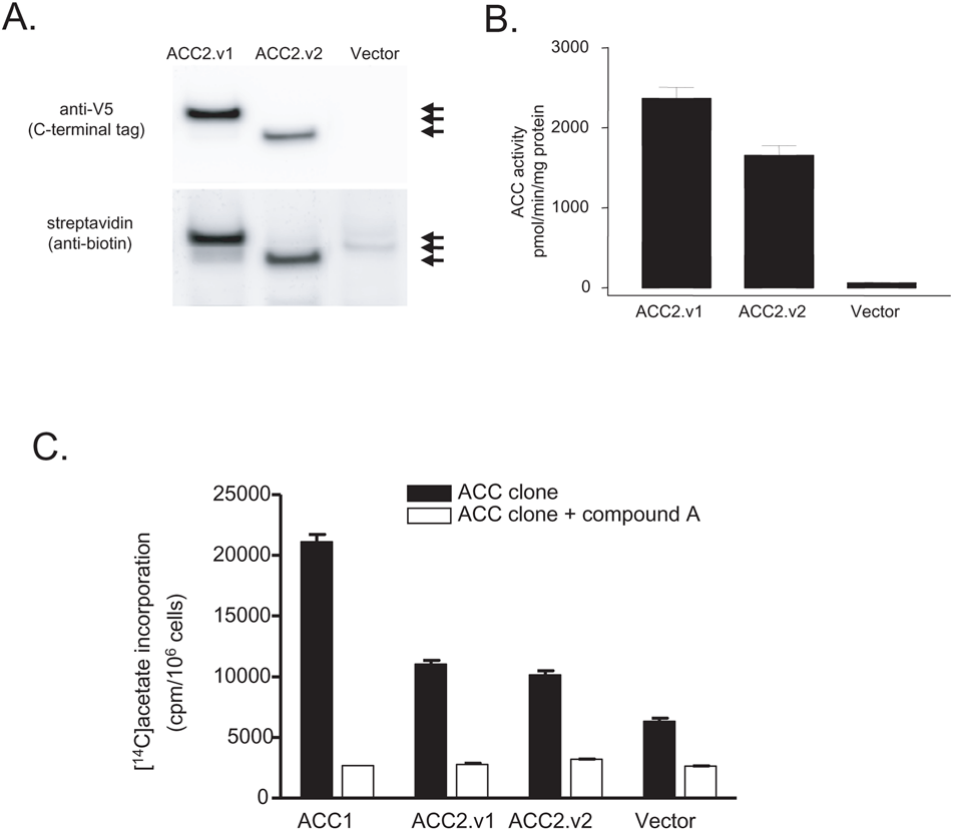
Expression and activities of ACC2 proteins. (A) Expression of recombinant ACC2.v1 and ACC2.v2 proteins in FM3A cells. Recombinant ACC proteins in cell lysates from FM3A cells overexpressing ACC isoforms were detected by Western blotting. Arrows mark the locations of ACC2.V1, ACC1, and ACC2.v2, from top to bottom. (B) ACC activities of recombinant ACC2.v1 and ACC2.v2 protein. ACC activities in FM3A cell lysates were measured by an *in vitro* ^14^CO_2_ fixation assay, measuring the incorporation of [^14^C]bicarbonate into [^14^C]malonyl-CoA. (C) DNL activity in FM3A cells overexpressing recombinant ACCs. DNL activities were measured as incorporated radio activities of [^14^C]acetate into cells in the presence of Simvastatin, a HMG-CoA reductase inhibitor. The non-specific[^14^C]acetate incorporation was determined in the presence of 10 µM a specific dual ACC1 and ACC2 inhibitor, labeled ‘compound A,’ and subtracted from total DNL activity in each FM3A clone.

To confirm the ACC2.v2 transcript isoform generates protein, we cloned ACC2.v2 and ACC2.v1 and overexpressed them in FM3A cells, a mammary carcinoma-derived cell line. Recombinant ACC2.v2 and ACC2.v1 were detected at expected molecular weights of ∼255 and ∼277 kDa, respectively and biotinylated in Western blotting (Figure 5A). We investigated ACC enzymological properties using the *in vitro* ^14^CO_2_ fixation assay using lysate from FM3A cells. Overexpression of either ACC2.v2 or ACC2.v1 increases ACC enzymatic activity >20-fold relative to vector alone (Figure 5B). Using purified recombinant human ACC1, ACC2.v1 and ACC2.v2, we found K_m_ values for acetyl-CoA, CO_2_ and ATP among ACC subtypes and variants, with K_m_ values for acetyl-CoA between 51 and 110 µM; for ATP between 44 and 110 µM; and for NaHCO_3_ between 2.9 and 6.5 mM (Table 1). Relative to ACC2.v1, ACC2.v2 showed 2 to 3-fold increases in K_m_ values for ACC2.v2 with respect to ATP and NaHCO_3_. Finally, we assayed *de novo* lipogenesis (DNL) activity in FM3A cells overexpressing recombinant ACC clones using [^14^C]acetate incorporation as a marker for DNL (Figure 5C). After overexpression, incorporation was first measured in the presence of a non-specific ACC inhibitor, labeled ‘compound A,’ as a control, and then without inhibitor. Overexpression of ACC1 increased cellular DNL activity almost 4-fold relative to vector alone. Cells expressing either ACC2.v2 or ACC2.v1 showed an approximately 2-fold relative increase in DNL.

**Table 1:** K_m_ values.

	K_m_ (µM)		
	Acetyl-CoA	ATP	NaHCO3
hACC1	51±3	54±5	6500±533
hACC2.v1	110±13	44±4	2900±193
hACC2.v2	94±13	110±7	6500±556

## Discussion

Our measurements of ACC1 and ACC2 transcript levels in rodent samples agree with published data [14], [23] showing high levels of ACC1 in adipose, high levels of ACC2 in heart and skeletal muscle tissues, and higher levels of ACC1 than ACC2 in liver. Also in agreement with published data [14], our microarray profiling of mouse brown adipose shows higher ACC2 expression than in mouse white adipose (data not shown). Human tissues show a very different expression pattern (Figure 1). Human adipose expresses ACC2 at levels four times higher than ACC1, a higher level than observed in human skeletal muscle. Human liver expresses more ACC2 than ACC1. Although ACC2 was first cloned from human adipose tissue [25], attention has focused on the role of ACC2 in fatty-acid oxidation in muscle tissue, and, to our knowledge, the high expression of ACC2 in human adipose has not previously been reported. Furthermore, microarray expression profiling in 53 dog and 65 rhesus monkey tissues (data not shown) show highest ACC2 levels in dog and rhesus adipose. Thus, ACC2 expression in primate and canine adipose is different than in rodent adipose, and may represent a species difference in fatty-acid regulation.

Although we have shown there are significant differences in ACC2 transcript and protein expression between rodent and human tissues, we cannot rule out the possibility that a part of the observed differences is due to variation in tissue dissection, RNA isolation, or sample pooling. However, adipose is a relatively homogeneous tissue, such that different dissection methods would not be expected to produce large changes in the cell-type composition of the isolated adipose tissue. Secondly, adipose samples collected from multiple vendors, for both individuals and pools of individuals, consistently show similar mRNA expression profiles that cluster together in a background of hundreds of other tissue samples (data not shown), suggesting that dissection and RNA isolation differences tend to produce relatively small effects on mRNA profiles.

We demonstrate the existence of a novel ACC2 isoform (ACC2.v2) that is expressed in human white adipose tissue and has enzymatic and DNL activity. The protein is smaller (∼255 kDa) than that of ACC1 (∼265 kDa) and the known ACC2 protein (ACC2.v1, ∼277 kDa) (Figure 5A, Supplemental Figures S1, S3). This form lacks the hydrophobic N-terminus found in the known ACC2 isoform, including the putative mitochondrial localization sequence but has all three catalytic domains essential for ACC enzyme activity. The ACC2.v2 mRNA accounts for the majority of ACC2 transcripts in testis, over 20% in adipose and approximately 5% in skeletal muscle. ACC2.v2 is also found in mouse and rat and is expressed at ratios similar to ACC2.v1 in these species (Figure 3), suggesting that the novel isoform is similarly regulated between species.

In human adipose, over 20% of the highly expressed ACC2 mRNA is in the form of ACC2.v2, which is expressed at levels comparable to ACC1. ACC2.v2 and ACC1 transcripts encode proteins that are 76% identical (87% similar), and the biotin carboxylation and carboxyl transferase regions are 84% and 81% identical (93% and 91% similar), respectively. The lack of the first coding exon of ACC2.v1 makes the ACC2.v2 protein sequence more similar to ACC1. Previous enzyme kinetics show only lower K_m_ for ACC1 than for ACC2 for acetyl-CoA and citrate [16], and our data suggest ACC2.v1 and ACC2.v2 have comparable ACC activity. All recombinant ACC isoforms tested, ACC1, ACC2.v1, and ACC2.v2, showed DNL activity in cells. However, given the moderate DNL activity of ACC2.v2 despite its level of protein expression, ACC2.v2 may not be as efficiently coupled to DNL pathway as ACC1. While cellular activity is also dependent on mRNA translational rates and phosphorylation states [23], [26]–[28], these expression and enzymatic and DNL activity results suggest that ACC2 isoforms, both ACC2.v1 and ACC2.v2, are capable of contributing to fatty-acid synthesis in human adipose tissue. This hypothesis would also help explain the observation that liver-specific ACC1 deficient mice showed hepatic DNL [7], and is consistent with the observation by Oh *et al.* [9] that ACC2^−/−^ mice showed not only increased fatty-acid oxidation but also decreased fat levels in adipose. Finally, our results also suggest ACC2 specific inhibition could result in different physiological effects in humans than in rodents.

## Materials and Methods

### Microarray data

Custom oligonucleotide microarrays were purchased from Agilent Technologies (Palo Alto, California). We designed these arrays to monitor the expression of 18,000 genes and associated alternate splicing events [29]. After alignment of 107,551 full-length human mRNA transcripts to the human genome, probes were designed to target every exon (60 mers) and every exon-exon junction (36 mers on 10 nucleotide T stilts) [24]. Poly[A]^+^ mRNA was amplified with a full-length amplification method using random-priming sequences to reproduce the entire transcript [24]. Fluorescent dye-labeling, hybridization conditions, and scanning were performed as previously [30]. Each amplified sample was hybridized twice against a common reference pool in a dye-swap experiment. Intensities shown are the mean of all probes for each transcript (113 for ACC1 and 120 for ACC2), after normalization using a common reference pool. Error bars estimate measurement error and not biological variation.

### Tissue samples

Human tissues and cell line samples were purchased as mRNA or total RNA (Clontech, Mountain View, California). Each tissue sample was pooled from multiple donors, typically 12. Human adipose tissue consisted of subcutaneous epithelial white adipose tissue. Mouse and rat tissues were purchased from Biochain (Hayward, California) and Clontech. Human protein samples were purchased from Biochain and Biopredic International (Rennes, France) as tissue lysate or frozen block, Male Sprague-Dawley rats from Japan SLC, (Hamamatsu, Japan) and C57Black/6J mice from CLEA Japan (Tokyo, Japan).

### Genomic analyses

Alignments to the human genome (NCBI Build 34, USCS hg16) were done with sim4 [31] for human ESTs and EST_GENOME [32] for mouse ESTs. FirstExon predictions [33] and human/mouse/rat/chicken conservation scores [34] were downloaded from UCSC genomic databases [35].

### RT-PCR

Supplemental Table S1 lists the RT-PCR primer sequences to monitor the known and novel first exons of ACC2. Reverse transcription-polymerase chain reaction (RT-PCR) amplification from tissue-specific mRNA or total RNA was performed as previously described [29]. The oligonucleotides were obtained from Qiagen (Valencia, California). Amplicons were subcloned into pCR2.1 using a TOPO-TA cloning kit (Invitrogen Corp., Carlsbad, California). Sequencing was performed by a commercial vendor (Lark Technologies Inc., Houston, Texas).

### Quantitative PCR

TaqMan primer and probe sequences used to monitor ACC1, ACC2.v1, and ACC2.v2 levels quantitatively in human and mouse tissues are shown in Supplemental Table S1. ACC2.v1 is the isoform defined by transcript NM_001093 and ACC2.v2 is the novel isoform defined here. TaqMan primer-probe reagents were obtained through the Applied Biosystems Assays-by-Design custom assay service (Foster City, CA). Probe sequences were designed to straddle a unique splice junction characteristic of each alternative splice form. TaqMan assays were performed on an ABI 7900 real time PCR instrument in 10 µl assays that were run in triplicate in a 384-well format optical PCR plate. The assays were calibrated with isoform-specific RT-PCR clones using the standard curve method [36]. Standard curves generated from plasmid clones were linear across at least six orders of magnitude, and all reported values derived for total tissue RNA fell within the range of these standard curves. RNA was converted to cDNA for TaqMan measurements using a commercially available kit from Applied Biosystems. All assays were normalized on a tissue-to-tissue basis by adding a constant amount of input total RNA into the RT reaction.

### Western blotting

Total protein samples were separated using 3–8% Tris-Acetate polyacrylamide gel electrophoresis (Invitrogen), and then transferred to PVDF membrane (Immobilon-P, Millipore, Billerica, Massachusetts). Membranes were blocked with Block Ace reagent (Dainippon Sumitomo Pharma Co., Ltd, Osaka, Japan/Snow Brand Milk Product Co. Ltd, Tokyo, Japan), and then incubated with HRP-conjugated streptavidin (Zymed Laboratories, South San Francisco, CA) or corresponding polyclonal antibodies followed by HRP-conjugated 2nd antibody according to the manufacturers (Pierce Biotechnology, Inc., Rockford, IL). Antibody dilutions are shown in Supplemental Table S2. Chemiluminescent detection was performed using SuperSignal West Femto Maximum Sensitivity Substrate (Pierce) and LAS-3000 luminescent image analyzer (FUJIFILM Corp., Tokyo, Japan). Polyclonal antibodies specific for ACC1, ACC2.v1 and ACC2.v2 were prepared by immunizing of polypeptides, shown in Supplemental Table S2, to New Zealand white rabbits. The anti-ACC2.v1 antibody was raised with N-terminal peptide for ACC2.v1 but not for ACC2.v2 (Supplemental Table S2).

### Cloning and expression

The full-length human ACC2.v1 cDNA was amplified by PCR from human skeletal muscle Marathon-Ready cDNA (Clontech) was used as the template using the following primer pair: forward, 5′-GCCTAGGTAATGGTCTTGCTTCTTTGTCTATCTTGTCTG-3′; reverse, 5′-ACCGGTGGTGGAGGCCGGGCTGTCCATG-3′. The amplified product was cloned into a pcDNA5/FRT/V5-His-TOPO vector (Invitrogen) with an added V5 epitope and His tags at the C-terminus, and was confirmed by sequencing. The plasmid was then digested with AvrII and PmeI, and the cDNA fragment was subcloned into the SpeI/EcoRV site of pEFcDNA3 (Invitrogen) vector. The ACC2.v2 cDNA was prepared by replacement of the ACC2.v1 specific N-terminus with that of ACC2.v2 by PCR using the ACC2.v1 cDNA as a template and the following primer pair: forward, 5′-GCCTAGGTAATGAGTCCTGCCAAGTGCAAGATCTGTTTCCCTGATCGCGAAGTAAAGCCGAGCATGTCGGGACTCCA-3′, reverse, 5′-GGCCGGTGCTTCCTCAAC 3′. The PCR product was then digested with AvrII/HindIII and exchanged with that of ACC2.v1 cDNA to construct a full-length ACC2.v2 cDNA clone. The ACC2.v1 and ACC2.v2 expression plasmids and pEFcDNA3 vector were introduced into FM3A (mouse mammary carcinoma) cells (RIKEN cell bank, #RCB0086) using Lipofectaimine 2000 (Invitrogen) or Effectene Transfection Reagent (Qiagen, Hilden, Germany), according to manufacturers' instructions. The FM3A cells were cultured in RPMI 1640 medium (Invitrogen) containing 10% fetal bovine serum (HyClone, Logan, UT) and 1 mg/ml G418 (Invitrogen) at 37°C in 5% CO_2_, 95% air to obtain stable transfectants. Cell lysate was prepared and subjected to ACC enzyme assay and Western blot analysis.

### Immunoprecipitation

The lysate of human adipose tissue was incubated with affinity-purified anti-ACC2.v2 antibody for 3 hours at 4°C. After adding Protein-A-agarose (Upstate, Charlottesville, VA), the incubation was continued for an additional hour. ACC protein in supernatant was precipitated by 35% saturated ammonium sulfate. The immunoprecipitate and precipitated supernatant were subject to Western blot analysis. Recombinant ACC2.v2 protein was added to the immunoprecipitation reaction as a marker of migration distance. The recombinant ACC2.v2 protein was expressed in FM3A cells and purified with HiTrap chelating column (GE Healthcare Piscataway, NJ).

### ACC enzyme assay

ACC activity was measured with a standard ^14^CO_2_ fixation assay which measures the incorporation of [^14^C]bicarbonate into [^14^C]malonyl-CoA with a 96-well plate format. Recombinant protein was incubated at 37°C for 40 minutes in a reaction mixture consisting of 50 mM HEPES (pH 7.5), 2 mM DTT, 20 mM MgCl_2_, 20 mM citrate, 4 mM acetyl-CoA, 5 mM ATP, 4 mM NaHCO_3_, and 0.08 mM [^14^C]NaHCO_3_ (50–62 mCi/mmol, GE Healthcare) at 80 µl/well, and the reaction was terminated by the addition of 20 µl 1 N HC1. Following evaporation of unreacted [^14^C]NaHCO_3_, radioactivity in the residue was measured with TopCount Microplate scintillation counter (PerkinElmer Inc., Wellesley, MA). For kinetic studies, the reaction was run at acetyl-CoA, ATP and NaHCO_3_ ranging from 3.9 to 2,000 µM, 8.7 to 5,000 µM and 80 to 8,080 µM, respectively. K_m_ values were determined by non-linear regression analysis of initial velocities, according to the standard Michaelis-Menten equation.

### Preparation of purified recombinant proteins

The FM3A cells expressing ACC2.v1 and ACC2.v2 were cultured in RPMI 1640, 10% fetal bovine serum, penicillin/streptomycin, and 0.5 mg/ml G418 at 37° C. and 5% CO_2_. The Sf9 cells were grown in Grace's Insect Cell Culture Medium (Invitrogen, Carlsbad, Calif.) containing 10% fetal bovine serum, 0.1 mg/ml kanamycin, and 0.1% PLURONIC® F-68 (Invitrogen, Carlsbad, Calif.) at 30° C and 5% CO_2_. Sf9 cells were collected by centrifugation and resuspended in the culture media at a cell density of 1×10^7^ cells/ml. The Sf9 cells were then incubated with the ACC1 expression virus stock solution (moi = approximately 5) for 1 hour at room temperature. The infected Sf9 cells were diluted with the culture media to cell density of 1×10^6^ cells, and then cultured for 3 days. The recombinant ACC proteins were purified with HiTrap chelating column (GE Healthcare Piscataway, NJ).

### De novo lipogenesis (DNL)

DNL activity in FM3A cells was determined by whole-cell incorporation of [^14^C]acetate into the lipids. FM3A cells were suspended in culture medium (RPMI1640+10% FBS containing 5 mM MEM sodium pyruvate and 1 µM Simvastatin, a HMG-CoA reductase inhibitor) in at the density of 0.1×10^6^ cell/50 µl and incubated at 37°C for 1 hour. After the incubation, 50 µl of culture medium were added to the cells and incubated another 1 hour in the presence or absence of 10 µM of a specific dual ACC1 and ACC2 inhibitor, labeled “compound A”. 18.5 kBq of [2-^14^C]acetate (2.07 GBq/mmol; GE Healthcare UK Ltd.) in 10 µl of culture medium was then added to the cells and the cells were incubated for additional 1 hour. At the end of the incubation, cells were harvested onto 96 well Unifilter GF/C (PerkinElmer, Inc.) using Unifilter 96 Harvester and washed with 4 times with ice-cold PBS^2−^. After dry up of the plate, 30 µl/well of MicroScint 0 scintillant (PerkinElmer, Inc.) were added to the plate and then radioactivity in the insoluble lipid fraction were counted by TopCount Microplate scintillation counter (PerkinElmer, Inc.).

## Supporting Information

Figure S1 Domains in the ACC protein structure [37], including the novel ACC2 isoform characterized here.
(0.03 MB PPT)

Figure S2 Alignment of the rat and human genomes at the novel exon and the start of the second exon (italics). There are no inserts or deletions after the ‘ATG’ start codon (purple) but many before this start site, suggesting that the protein (underlined) does not extend in the upstream direction of this ATG.
(0.02 MB PPT)

Figure S3 Detection of endogenous ACC2.v2 protein in human adipose samples. Supernatant and immunoprecipitate from a human adipose lysate was probed with streptavidin (anti-biotin) and with an anti-ACC2.v2 antibody (Supplemental Table 1) (columns A and B). The supernatant probed with streptavidin shows a band at the expected ACC2.v1 location and at the ACC1/ACC2.v2 location (column A, upper). The immunoprecipitate probed with the ACC2.v2-specific antibody (column B, lower) shows a band at the predicted ACC.v2 location, suggestive of endogenous ACC2.v2 protein expression. Columns C and D repeat the experiment after adding recombinant ACC2.v2 protein. The band location in the lower C and D panels marks the ACC2.v2 migration distance. Arrows indicate the predicted ACC2.v1, ACC1, and ACC2.v2 locations (top to bottom, respectively).
(0.23 MB PPT)

Table S1 TaqMan probe sequences
(0.04 MB DOC)

Table S2 Antibodies used
(0.03 MB DOC)
