# Correction: ACC2 Is Expressed at High Levels in Human White Adipose and Has an Isoform with a Novel N-Terminus

**DOI:** 10.1371/annotation/c921dfce-9702-4632-abbe-f13d62fdfde0

**Published:** 2009-02-07

**Authors:** John C. Castle, Yoshikazu Hara, Christopher K. Raymond, Philip Garrett-Engele, Kenji Ohwaki, Zhengyan Kan, Jun Kusunoki, Jason M. Johnson

There was an error in the title of the article, which omitted the word "in." The correct title should read: ACC2 Is Expressed at High Levels in Human White Adipose and Has an Isoform with a Novel N-Terminus. This also affects the article's citation.

