# Supplementary material for: ACC2 Is Expressed at High Levels Human White Adipose and Has an Isoform with a Novel N-Terminus
Source: PLoS One. 2009 Feb 3;4(2):e4369. doi: 10.1371/journal.pone.0004369 (PMC2629817; doi:10.1371/journal.pone.0004369)
Supplement: Figure S1 — Domains in the ACC protein structure [37], including the novel ACC2 isoform characterized here. (0.03 MB PPT) [file pone.0004369.s001.ppt]

## Slide 1
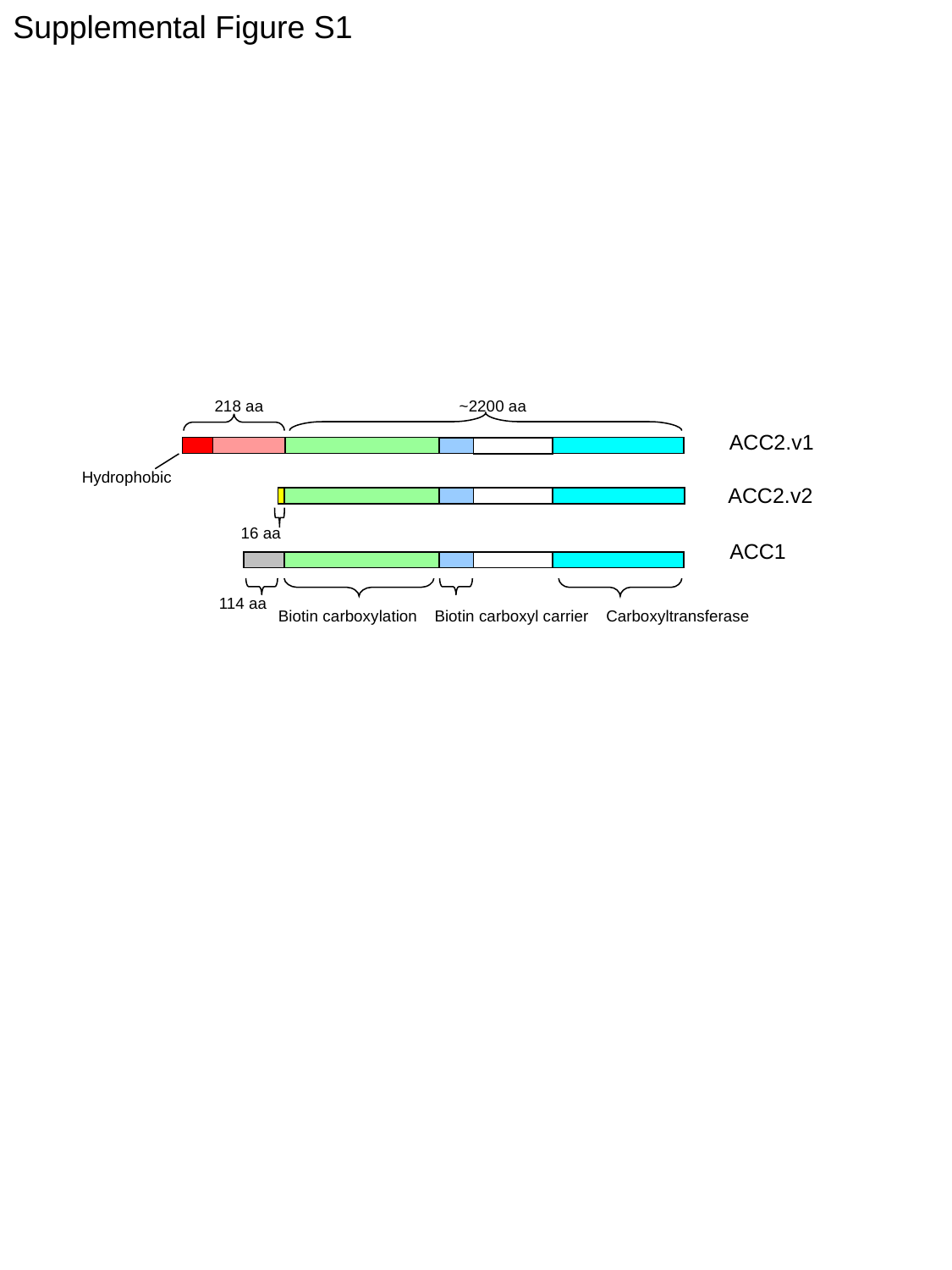

Supplemental Figure S1
218 aa
~2200 aa
ACC2.v1
Hydrophobic
ACC2.v2
16 aa
ACC1
114 aa
Biotin carboxylation
Biotin carboxyl carrier
Carboxyltransferase
