# Supplementary material for: ACC2 Is Expressed at High Levels Human White Adipose and Has an Isoform with a Novel N-Terminus
Source: PLoS One. 2009 Feb 3;4(2):e4369. doi: 10.1371/journal.pone.0004369 (PMC2629817; doi:10.1371/journal.pone.0004369)
Supplement: Figure S2 — Alignment of the rat and human genomes at the novel exon and the start of the second exon (italics). There are no inserts or deletions after the ‘ATG’ start codon (purple) but many before this start site, suggesting that the protein (underlined) does not extend in the upstream direction of this ATG. (0.02 MB PPT) [file pone.0004369.s002.ppt]

## Slide 1
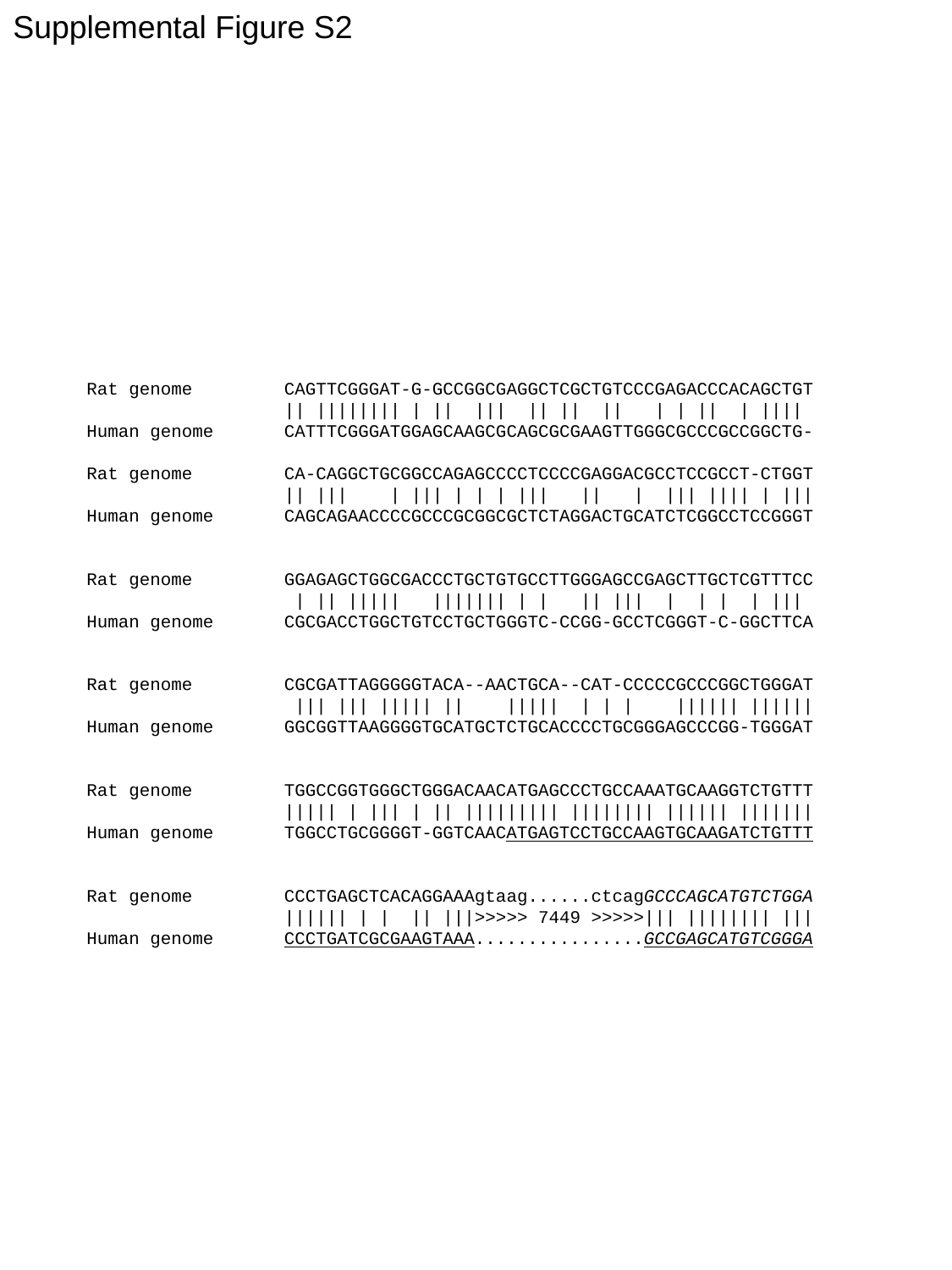

Supplemental Figure S2
Rat genome 	CAGTTCGGGAT-G-GCCGGCGAGGCTCGCTGTCCCGAGACCCACAGCTGT
 	|| |||||||| | || ||| || || || | | || | ||||
Human genome	CATTTCGGGATGGAGCAAGCGCAGCGCGAAGTTGGGCGCCCGCCGGCTG-
Rat genome 	CA-CAGGCTGCGGCCAGAGCCCCTCCCCGAGGACGCCTCCGCCT-CTGGT
	|| ||| | ||| | | | ||| || | ||| |||| | |||
Human genome 	CAGCAGAACCCCGCCCGCGGCGCTCTAGGACTGCATCTCGGCCTCCGGGT
Rat genome 	GGAGAGCTGGCGACCCTGCTGTGCCTTGGGAGCCGAGCTTGCTCGTTTCC
	 | || ||||| ||||||| | | || ||| | | | | |||
Human genome	CGCGACCTGGCTGTCCTGCTGGGTC-CCGG-GCCTCGGGT-C-GGCTTCA
Rat genome 	CGCGATTAGGGGGTACA--AACTGCA--CAT-CCCCCGCCCGGCTGGGAT
	 ||| ||| ||||| || ||||| | | | |||||| ||||||
Human genome 	GGCGGTTAAGGGGTGCATGCTCTGCACCCCTGCGGGAGCCCGG-TGGGAT
Rat genome 	TGGCCGGTGGGCTGGGACAACATGAGCCCTGCCAAATGCAAGGTCTGTTT
 	||||| | ||| | || ||||||||| |||||||| |||||| |||||||
Human genome 	TGGCCTGCGGGGT-GGTCAACATGAGTCCTGCCAAGTGCAAGATCTGTTT
Rat genome 	CCCTGAGCTCACAGGAAAgtaag......ctcagGCCCAGCATGTCTGGA
 	|||||| | | || |||>>>>> 7449 >>>>>||| |||||||| |||
Human genome 	CCCTGATCGCGAAGTAAA................GCCGAGCATGTCGGGA
