# Supplementary material for: ACC2 Is Expressed at High Levels Human White Adipose and Has an Isoform with a Novel N-Terminus
Source: PLoS One. 2009 Feb 3;4(2):e4369. doi: 10.1371/journal.pone.0004369 (PMC2629817; doi:10.1371/journal.pone.0004369)
Supplement: Figure S3 — Detection of endogenous ACC2.v2 protein in human adipose samples. Supernatant and immunoprecipitate from a human adipose lysate was probed with streptavidin (anti-biotin) and with an anti-ACC2.v2 antibody (Supplemental Table 1) (columns A and B). The supernatant probed with streptavidin shows a band at the expected ACC2.v1 location and at the ACC1/ACC2.v2 location (column A, upper). The immunoprecipitate probed with the ACC2.v2-specific antibody (column B, lower) shows a band at the predicted ACC.v2 location, suggestive of endogenous ACC2.v2 protein expression. Columns C and D repeat the experiment after adding recombinant ACC2.v2 protein. The band location in the lower C and D panels marks the ACC2.v2 migration distance. Arrows indicate the predicted ACC2.v1, ACC1, and ACC2.v2 locations (top to bottom, respectively). (0.23 MB PPT) [file pone.0004369.s003.ppt]

## Slide 1
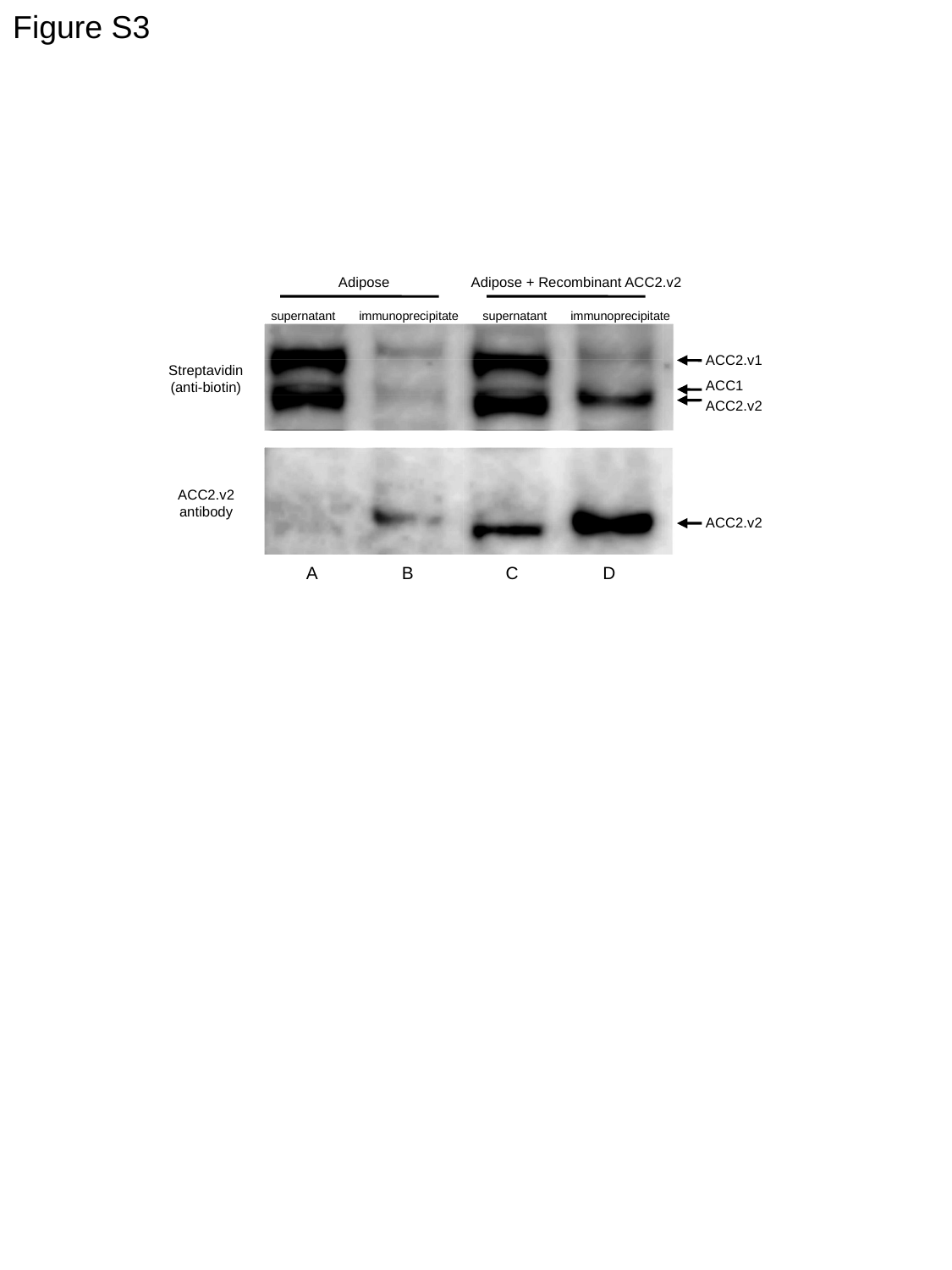

Figure S3
	Adipose
	Adipose + Recombinant ACC2.v2
	supernatant 	 immunoprecipitate
	supernatant 	 immunoprecipitate
ACC2.v1
Streptavidin
(anti-biotin)
ACC1
ACC2.v2
ACC2.v2
antibody
ACC2.v2
A
B
C
D
