# Supplementary material for: ACC2 Is Expressed at High Levels Human White Adipose and Has an Isoform with a Novel N-Terminus
Source: PLoS One. 2009 Feb 3;4(2):e4369. doi: 10.1371/journal.pone.0004369 (PMC2629817; doi:10.1371/journal.pone.0004369)
Supplement: Table S1 — TaqMan probe sequences (0.04 MB DOC) [file pone.0004369.s004.doc]

**Supplemental Table S1. TaqMan probe sequences.**

| Species | Target | Forward Primer | Reverse Primer | probe |
| --- | --- | --- | --- | --- |
|  | ACC1 | Hs00167385_m1; ABI Assay-on-demand |  | CCCTCACCCAACCCAAAAAGGTCAG |
| Huma TaqMan | ACC2.v1 | GACCACAGGTGAAGCTGAGA | GTGTTCCCGTCCCCTCTTC | ACATGCTCGGCCTCATAG |
|  | ACC2.v2 | AAGTGCAAGATCTGTTTCCCTGAT | CAGGTGGAGTCCCGACATG | CTCGGCTTTACTTCGCG |
|  | ACC2 both | Hs00153715_m1 ABI Assay-on-demand |  | CACTATGAGGCCGAGCATGTCGGGA |
| Human RT-PCR | ACC2.v1 | TTATCTGACCACAGGTGAAGCTGAGA | GCTCCGGAAGTTTAGGGTTTTCTAAAG | NA |
|  | ACC2.v2 | CAAGTGCAAGATCTGTTTCCCTGATCG | CCGGAAGTTTAGGGTTTTCTGAAGCAT | NA |
|  | ACC1 | CAGGATGGTTTGGCCTTTCAC | TTTCTTTCTGTCTCGACCTTGTTTTACT | ATGAGGTCCAGCATGTCC |
| Rat TaqMan | ACC2.v1 | ATCCTGAGTCTCACACACCTACT | CGGCCTCTCTTCACCAGATG | ATGAGGCCCAGCATGTC |
|  | ACC2.v2 | GCCAAATGCAAGGTCTGTTTCC | CGGCCTCTCTTCACCAGATG | ATGCTGGGCTTTCCTG |
|  | ACC2 both | GCTTTGGAGGCAACAGGGTTAT | CCGCAGCGATACCATTATTGG | CGAGACGGTGCTCATC |
|  | ACC1 | AAACTGCAGGTATCCCAACTCTTC | CTGTGGAACATTTAAGATACGTTTCGAAAA | TCGAAGACCACTGCCACTC |
| Mouse TaqMan | ACC2.v1 | GACCACAGGTGAAGCTGAGA | GTGTTCCCGTCCCCTCTTC | ACATGCTCGGCCTCATAG |
|  | ACC2.v2 | AAGTGCAAGATCTGTTTCCCTGAT | CAGGTGGAGTCCCGACATG | CTCGGCTTTACTTCGCG |
|  | ACC2 both | GACGCCCGAGGATCTGAAG | GGGACAGGGACGTACTGATC | CAACGCAGAGTACATCAA |
