# Supplementary material for: ACC2 Is Expressed at High Levels Human White Adipose and Has an Isoform with a Novel N-Terminus
Source: PLoS One. 2009 Feb 3;4(2):e4369. doi: 10.1371/journal.pone.0004369 (PMC2629817; doi:10.1371/journal.pone.0004369)
Supplement: Table S2 — Antibodies used (0.03 MB DOC) [file pone.0004369.s005.doc]

**Supplemental Table S2. Antibodies used.**

| Antibody | Target | Target Region | Dilution (fold) |
| --- | --- | --- | --- |
| Streptavidin-HRP | ACC1,  ACC2.v1 and ACC2.v2 | Biotin | 8,000 ~ 12,500 |
| Anti-V5 | Recombinant ACC1, ACC2.v1 and ACC2.v2 | V5 epitope | 8,000 |
| Anti-ACC1 | ACC1 | Human ACC1 a.a.1259-1276 (SPPQSPTFPEAGHTSLYD) | 150 ~ 300 |
| Anti-ACC2.v1 | ACC2.v1 | Human ACC2.v1 a.a.95-108 (SRQKPPRNPLSSSD) | 2,000 |
| Anti-ACC2.v2 | ACC2.v2 | Human ACC2.v2 a.a.1-16  (MSPAKCKICFPDREVK) | 150 |
